# Smart, automated junctional tourniquets leveraging AI-driven ultrasound guidance

**DOI:** 10.1038/s41598-026-37467-1

**Published:** 2026-02-01

**Authors:** Sofia I. Hernandez Torres, Theodore Winter, Isiah Mejia, Carlos Bedolla, Benjamin Alexander, James P. Collier, Michael D. Lopez, Eric J. Snider

**Affiliations:** https://ror.org/02jxznk77 grid.461685.8 0000 0004 0467 8038 U.S. Army Institute of Surgical Research, Organ Support and Automation Technologies Group, JBSA Fort Sam Houston, San Antonio, TX 78234 USA

**Keywords:** Engineering, Health care, Medical research

## Abstract

Tourniquets are commonly used devices for hemorrhage control; however, their effectiveness is reduced in anatomical junctions such as the neck and inguinal region. Junctional tourniquets specifically require precise placement to be effective. This precision can be enabled with ultrasound technology to help locate and occlude the major vessels in the junctional regions properly. However, interpretation of ultrasound requires highly skilled personnel, who may not necessarily be available in emergency situations. To overcome this hurdle, we have developed two ultrasound-enabled, AI-driven junctional tourniquet prototypes. AI models can aid in guiding the end-user to the correct location and determine occlusion during and after pressure application. Proof-of-concept functionality of the developed prototypes integrated with AI models was successfully tested in a durable, ultrasound-compatible femoral tissue phantom and compared against commercially available tourniquet devices. Overall, time to occlusion was comparable between the tourniquet prototype designs and traditional junctional tourniquets, while each AI model achieved high performance metrics for this application. As such, the combination of AI and ultrasound can prove to be a viable solution to prevent further hemorrhaging at the point of injury.

## Introduction

Hemorrhage remains one of the leading causes of preventable death for both civilian and military trauma^1,2^. The first step in preventing death from a hemorrhagic injury is stopping the bleeding to minimize overall blood loss. This can be done for different injury types using a wide range of approaches, such as tourniquets, wound packing, direct pressure or surgical intervention^3,4^. Tourniquets in particular have proven to be highly effective for extremity hemorrhage control, especially during combat casualty care^5–7^. However, tourniquets for application at key anatomical or truncal junctions – inguinal or femoral, neck or subclavian, and aortic – have been less effective^8^.

Currently, there are several commercially available junctional tourniquets that utilize pressure points for hemorrhage control. These devices are designed to be placed at pressure point to apply and maintain compression on the vessels. Currently, there are four devices that have been approved by the Food and Drug Administration (FDA): Combat Ready Clamp (CRoC), Junctional Emergency Treatment Tool (JETT), SAM^®^ Junctional Tourniquet (SJT), and Abdominal Aortic and Junction Tourniquet (AAJT)^9–12^. Each has a different placement mechanism and all are recommended to be deployed for less than four hours, depending on the placement site. However, studies have shown that these devices are cumbersome and time-consuming to place^13,14^, and they also have a high failure rate for controlling hemorrhage in both training and battlefield scenarios^15,16^. Current junctional tourniquets are associated with high malfunction rates during initial application^17,18^, potentially due to the higher skill-threshold required. Furthermore, they often slip during transport^19^, diminishing device reliability. Precise and secure positioning is primarily important as compression must occur where the blood vessels align with a solid, bony surface beneath for hemorrhage control. Imprecise placement can lead to tourniquet failure or tissue damage from overtightening at the incorrect locations.

Recent developments focus on junction hemorrhage control via novel tourniquet technology, wound packing materials, and manual pressure point occlusion. A recent study evaluated the effectiveness of the Life Saving Tourniquet (LST) which combines features of the current commercial products^20^. In more than 30 human volunteers, a 96% success rate at achieving full vessel occlusion was reached. However, the tourniquet still required precise and manual positioning and was not tested in the hands of novice end-users. Wound packing techniques create pressure inside the bleeding wound by filling the cavity to control the bleeding with hemostatic dressing or mini-sponge material^21–23^. While these can be effective at controlling hemorrhage coming from a venous vessel, it can become unreliable against hemorrhage coming from a major arterial source as the packing material tends to absorb blood without stopping the hemorrhage, causing a wicking effect^21,24^. The manual pressure technique involves applying pressure to a major artery, proximal to the hemorrhage site, against a bony surface. Manual pressure point occlusion can be highly effective but has been shown to be challenging to maintain for a prolonged period of time and should only be used as a temporary solution^25,26^, which would be ineffective in prolonged field care situations. In addition, successful manual hemorrhage control requires extensive training and knowledge of the junctional site, which is also likely limited in emergency situations.

In summary, junctional hemorrhage control remains a mostly unsolved problem in the pre-hospital setting as current solutions and technologies are inadequate to properly achieve hemorrhage control^8^. As such, there is a need for improved approaches, and we postulate that the integration of automation technology through medical imaging and artificial intelligence (AI) can improve junctional tourniquet use and effectiveness. One of these automation avenues relies on the use of an ultrasound (US) transducer as a tool to apply the necessary pressure for controlling junctional hemorrhage while visualizing the underlying anatomy, ensuring proper placement over time^27^. The ability to track vessel patency status while compressing to occlude flow can provide continuous monitoring allowing for immediate identification of slippage. While this provides evidence that US can aid with junctional applications, US image interpretation requires highly trained personnel that may not be present in the pre-hospital setting where hemorrhage control is most critical.

To address these shortcomings, an AI-enabled device may be able to improve both casualty outcome and user experience by supporting US image interpretation^28^. The AI models can provide object detection (OD) outputs which can generate bounding boxes around anatomical features of interest such as vessels, nerves, or osseous structures to aid the user with compression alignment. Object detection AI models have been successfully used for biomedical applications to identify anatomical structures from ultrasound images in liver disease diagnostics^29^, thyroid nodule detection^30^, and lung feature recognition^31^. We have previously shown that object detection models can be a valuable tool for guiding correct image capture for ultrasound triage assessment^32^, a similar application to US-driven junctional tourniquet guidance to the correct hemorrhage control site.

Another area of junctional hemorrhage control that can benefit from AI automation is the application of pressure necessary to achieve and sustain vessel occlusion. Tourniquet overtightening has been linked to irreversible nerve or muscle damage while lack of occlusion can continue to exacerbate the hemorrhage injury^33^. As such, AI models to identify proper occlusion paired with US-driven prototype tourniquet devices capable of mechanical actuation could automate this process. For tracking occlusion, a more traditional classification AI model can be developed for distinguishing between compressed and non-compressed vessels. US image interpretation has used AI models for detecting vascular diseases such as deep vein thrombosis^34^ and evaluating blood flow return after vessel compression^26,35^. Through a preliminary study, it was concluded that classification AI models were suitable for tracking vessel occlusion by interpreting US color Doppler images, as confirmed by fluid flow^27^. However, these models were not evaluated in real-time nor were they integrated with portable US technology and mechanical actuation to automate the junctional occlusion process fully. For battlefield and remote settings, with restricted power consumption, relying on color Doppler US may not be practical as it can limit the duration of device operability^36^ especially for portable, handheld systems.

In this effort, we developed initial prototype tourniquet devices for automated ultrasound-guided junctional hemorrhage control. Multiple prototypes were fabricated based on set design criteria and paired with trained object detection and image classification AI models, as well as mechanical actuation to streamline device guidance to the proper location and automate occlusion. Prototypes were tested in various tissue phantom models to highlight proof-of-concept design features and feasibility of automated junctional hemorrhage control. Fully integrated prototype testing was conducted with the goal of identifying the anatomical feature alignment and subsequently triggering mechanical actuation to achieve hemorrhage control.

## Results

Two junctional tourniquet prototype devices were developed for integration with AI models providing anatomical guidance and occlusion tracking. Conceptually, each design was built for handheld manual movement to the proper occlusion site driven by guidance AI model feedback, securement of the prototype at the proper site, and automated occlusion with AI paired with *z*-directional actuation. For clarity, a goal of 90% flow reduction was set as the definition for occlusion success when assessing prototype performance. The results shown here first characterize duration of device assembly, securement, and occlusion for each prototype at the different anatomical locations, relative to commercial tourniquet devices. Then, pairing with the AI models for evaluating the effectiveness of anatomical guidance and occlusion accuracy. Lastly, how the AI-integrated prototype devices perform in proof-of-concept testing of the entire automated process.

### Junctional Tourniquet Prototype Characterization

**Fig. 1:**
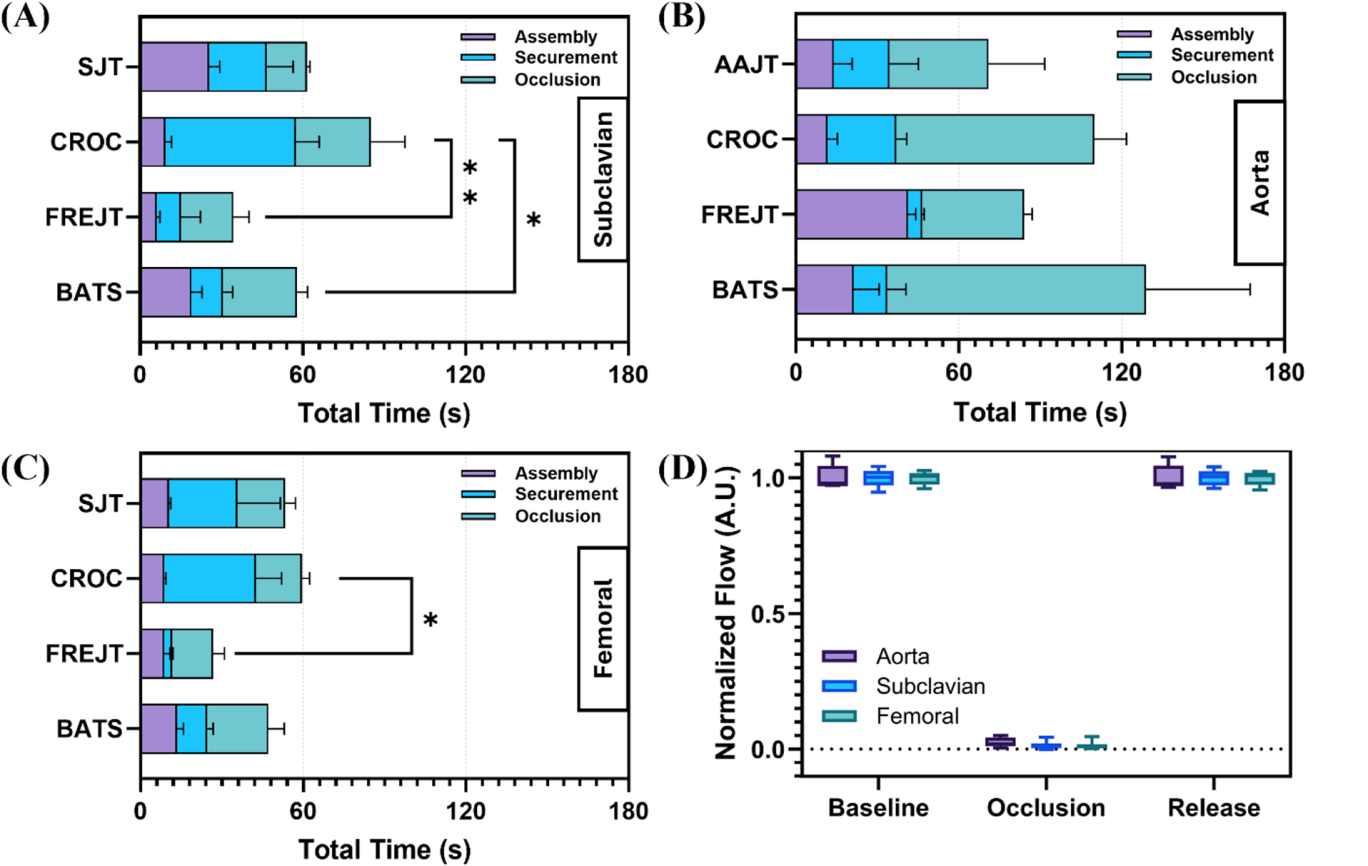
Summary of testing performance for each prototype and commercial tourniquet for (**A**) subclavian, (**B**) aorta, and (**C**) femoral placements. Times are shown as average for the assembly, securement, and occlusion period in stacked bar plots, with error bars denoting the standard deviation for each (*n* = 3 replicates) run that achieved proper placement. (**D**) Validation of flow rate changes for all sites during baseline, occlusion, and release of pressure. Statistically significant differences between overall tourniquet times were determined by ordinary one-way ANOVA, post-hoc Holm-Šídák test (* for p-values < 0.05; ** for p-values < 0.01). Flow rates are shown in arbitrary units with the height of the box representing the first and third quartile and error bars denoting range of values.

**Fig. 2:**
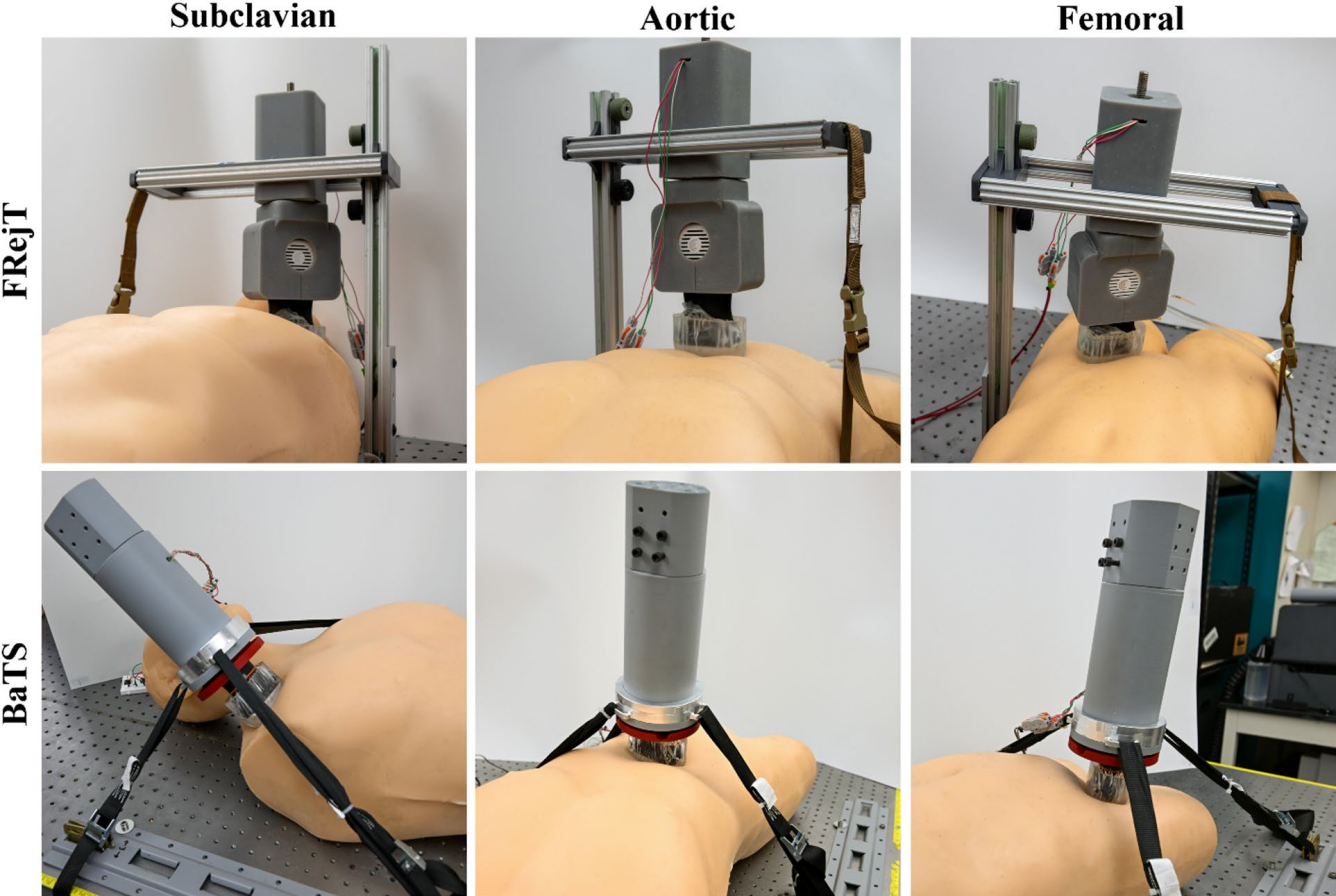
Representative images of FRejT (First row) and BaTS (Second row) placement at the Subclavian (First column), Aortic (Second column), and Femoral (Third column) sites in the commercial tissue phantom.

Testing was conducted to evaluate the prototyped junctional tourniquets compared against commercially available junctional tourniquets for time to fully assemble, secure at location, and reach occlusion. This was done for three junctional tourniquet sites: (i) subclavian, (ii) aortic and (iii) femoral regions. The assembly and securement times were recorded in a commercial tissue phantom that mimicked human anatomical contour, while the occlusion times were recorded using the custom tissue phantoms, perfused and pressurized to a mean pressure of 75 mmHg with an active bleed. The custom tissue phantoms were capable of being compressed to occlusion, with flow reducing to under 5% of initial flow rate for each anatomical location (1.6 ± 1.2% subclavian; 2.4 ± 1.6% aorta; 1.3 ± 1.2% femoral; *n* = 12 runs) but recovering to full flow rate after releasing the compression (Fig. 1D). Both tourniquet designs, the Frame Reinforced Junctional Tourniquet (FRejT, Fig. 2, first row) and Base and Tightening Straps design (BaTS, Fig. 2, second row), were used at all anatomical sites along with the commercial CRoC tourniquet (not shown). In addition, the AAJT (not shown) was used at the aortic site while the SJT device (not shown) was used at the subclavian and femoral sites so that two commercial products were used for each site.

Starting with the subclavian site, the overall fastest device to reach occlusion was FRejT at 34.3 ± 12.5s which was significantly quicker than CRoC, the slowest device at 85.0 ± 19.2s (*p* = 0.0084, Fig. 1A). BaTS took 51.05 ± 12.2s, which was significantly quicker than the CROC device (*p* = 0.049), but the BaTS prototype experienced a slip during placement. Despite having the longest time to reach occlusion among devices tested, CRoC managed to have one of the fastest assembly times at 9.40 ± 2.30s. For the aortic site, the AAJT reached occlusion in the shortest amount of time (73.6 ± 14.0s) with occlusion time phase taking the longest (Fig. 1B). FRejT was the most consistent of the devices tested across replicate runs with the quickest securement times. The BaTS prototype took the longest to reach occlusion at 129.0 ± 44.0s for the aortic site. Lastly, at the femoral site, FRejT was quickest at 26.9 ± 5.5s and was significantly quicker than CRoC at 59.6 ± 7.4s (*p* = 0.038, Fig. 1C). Overall, at the femoral occlusion site, all tourniquets were quicker to assemble, secure and occlude, and replicates were more consistent.

### Guidance and occlusion AI model performance

**Fig. 3:**
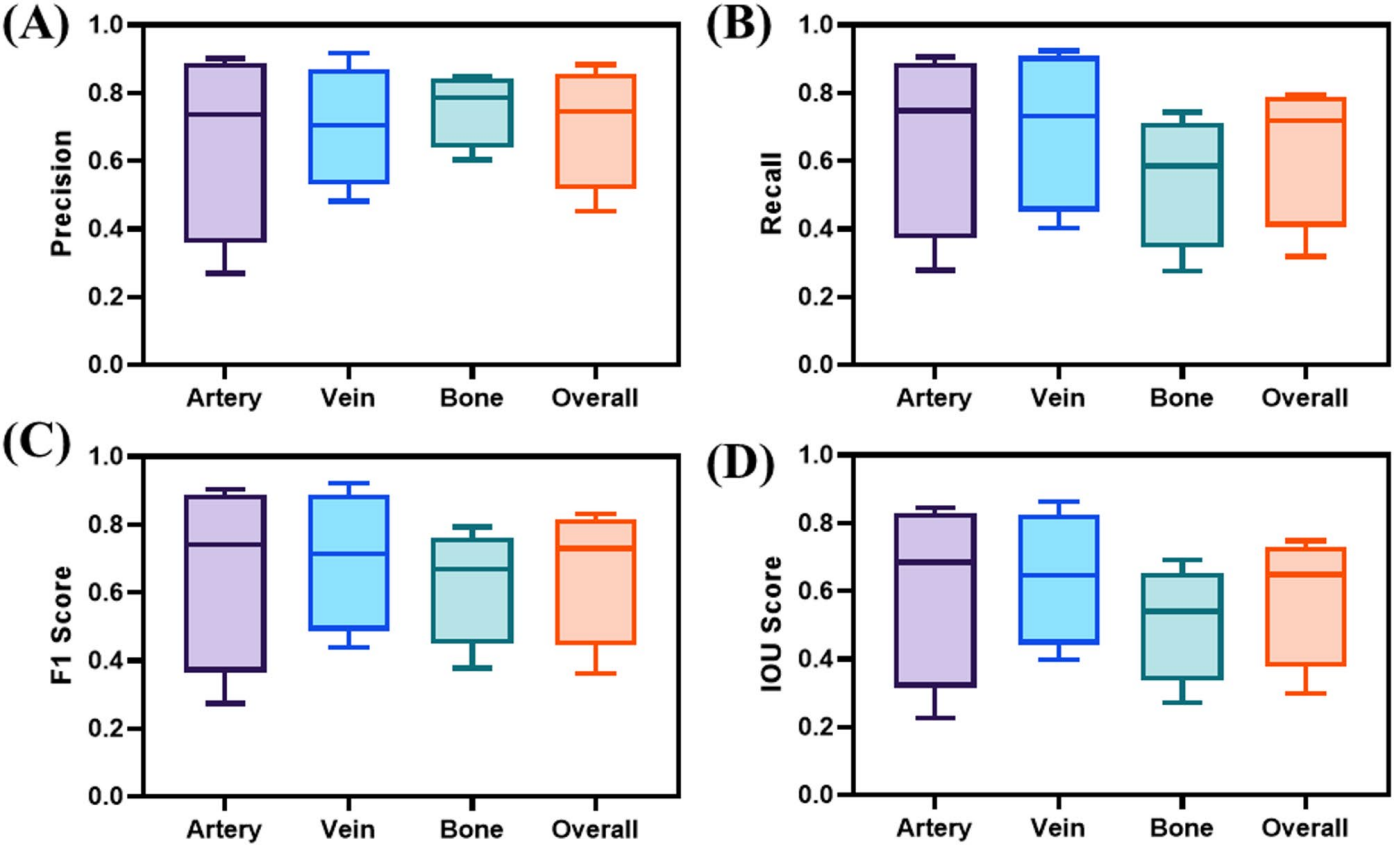
Summary of guidance AI model performance metrics. Anatomical guidance object detection model performance metrics on blind test subjects for (**A**) Precision, (**B**) Recall, (**C**) F1 Scores, and (**D**) IOU. Artery, Vein, and Bone are shown separately alongside overall performance metric scores. Results are shown as box and whisker plots, with error bars indicating minimum and maximum values while the length of the box indicates interquartile range and median value is denoted by the line (*N* = 4 US video captures).

Next, guidance AI models were trained for tracking anatomical features for the automated junctional tourniquet application, and their validation and blind test performance were evaluated. The guidance object detection models achieved an average precision score of 0.876 ± 0.042 on validation data among all three classes with artery performing the highest with a precision of 0.922, while vein received the lowest precision of 0.840. However, guidance evaluation metrics regressed when inference was performed on blind US holdout testing data from the femoral site at different views and angles, yielding an average precision of 0.706 ± 0.183 (Fig. 3A), recall of 0.638 ± 0.220 (Fig. 3B), F1 score of 0.663 ± 0.208 (Fig. 3C) and intersection over union (IOU) scores of 0.586 ± 0.20 (Fig. 3D) across all three classes. Overall, object detection models performed better on more curated data where the US transducer was directly over the inguinal crease, and, thus, all objects of interest were clear, compared to images where the transducer was offset or angled relative to the anatomical features of interest.

**Fig. 4:**
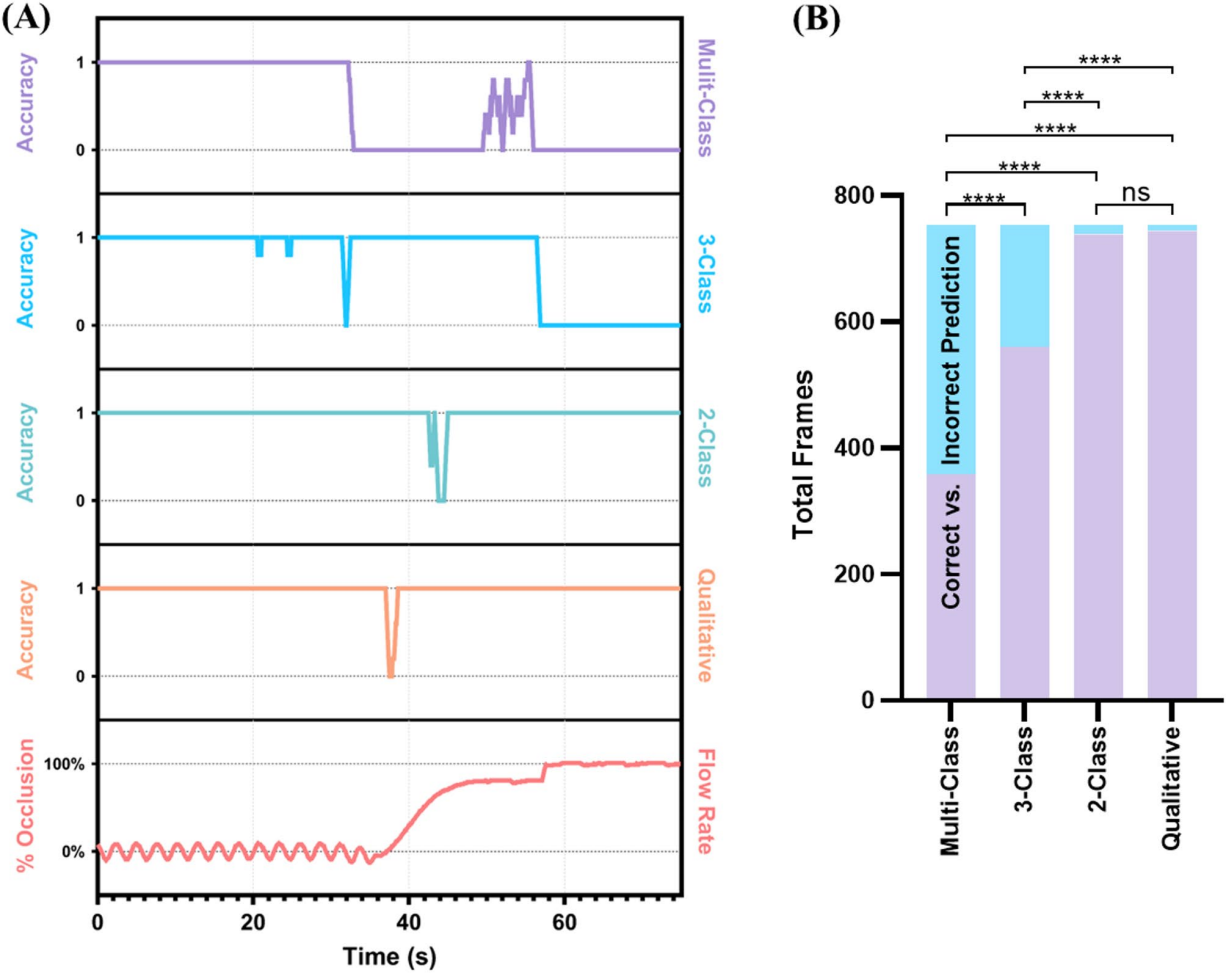
Summary of occlusion AI model performance metrics. (**A**) Comparison of performance accuracy for different occlusion AI classification model configurations. Results are averaged across data capture experiment starting at baseline, progressive pressure, and reaching full occlusion, as indicated by the bottom “Flow Rate” figure panel. Average accuracies are shown for the multi-class, three-class, two-class, and qualitative image classification models. (**B**) Summary of correct and incorrect predictions across all test images (*N* = 754 frames) for each occlusion AI model. Statistical differences between occlusion AI models were determined by McNemar test with statistically significant (**** denotes p-value < 0.0001) and not significant results shown (ns).

A second AI model was developed for tracking occlusion state of the femoral vessels and monitoring junctional hemorrhage control. Different training configurations were characterized for this task, ranging from distinguishing 7 classes of incremental occlusion levels to simple binary classification models tracking qualitative vessel occlusion based on US image assessment of lumen visibility. The accuracy of each occlusion classification model increased as the task being performed became simplified (Fig. 4A). The original seven-class model returned a percentage value of vessel occlusion and yielded a 0.479 ± 0.500 accuracy. The multiclass model was consistently only accurate at detecting images where the site was at a baseline flow rate and inaccurate at distinguishing distinct occlusion levels, highlighting the need to simplify the classification AI for occlusion. Next, a three-class model that returned whether the site was at baseline flow, in-progress of being occluded, or occluded (> 90% flow reduction) saw a significant accuracy improvement of 0.744 ± 0.437 (p-value < 0.0001) and was successful at detecting the first two classes but failed to consistently identify full occlusion. For the next iteration of occlusion models a binary approach was taken, wherein anything under 50% of base arterial flow rate was categorized as a baseline image while any higher occlusion levels were categorized as occluded. Accuracy significantly improved to 0.980 ± 0.140 (p-value < 0.0001) but the model struggled to distinguish between the two classes at or near the 50% occlusion level, resulting in occlusion levels up to 60% being identified as baseline. Lastly, an alternative two-class classification model with classes qualitatively split between baseline and occlusion based on vessel lumen being visible in the US image resulted in a slightly better accuracy of 0.987 ± 0.115 but this difference was not statistically significant when compared to the 2-class model (p-value = 0.317). However, the incorrect predictions from the model occurred earlier during pressure application to the blood vessel, allowing for accurate, earlier detection of vessel compression.

**Fig. 5:**
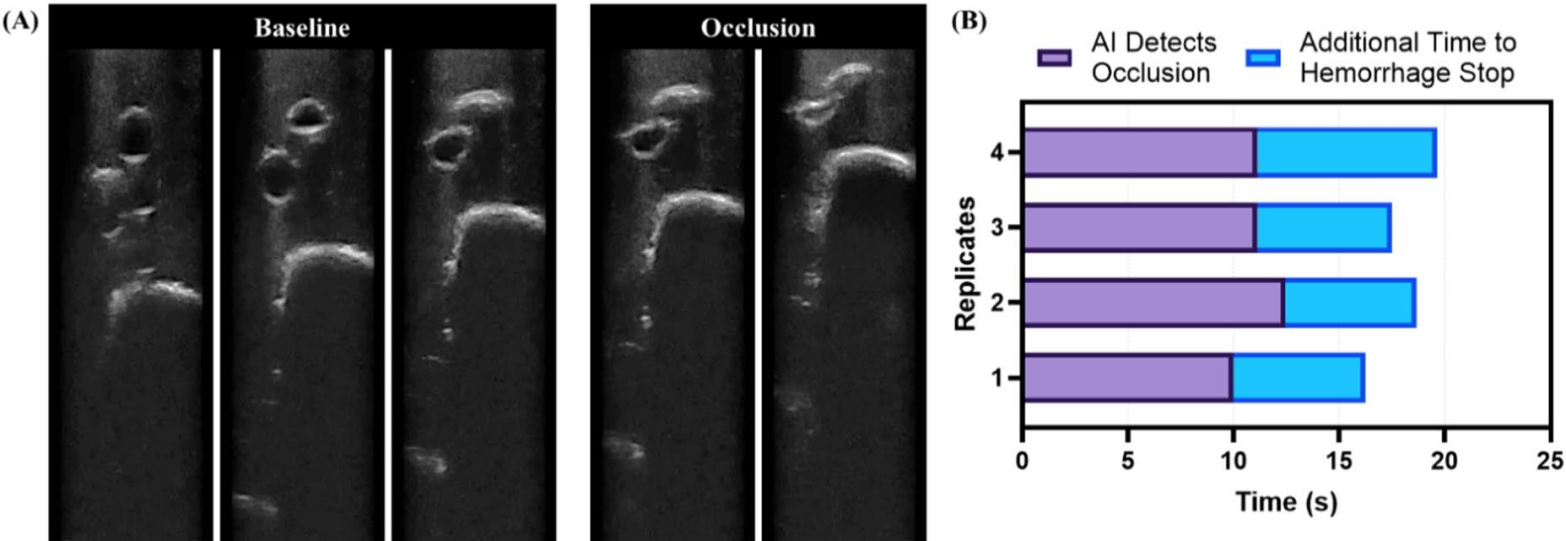
Timing to reach occlusion. (**A**) Representative ultrasound images from JUGO phantom categorized as “baseline” or “occlusion” based on the qualitative split criteria. Image brightness and contrast were adjusted for visualization purposes. (**B**) Summary of times for detecting occlusion by the AI classification model and the additional time to reach objective occlusion, defined as 90% flow reduction. Four individual replicates are shown to highlight the performance consistency.

As such, the qualitative approach was selected and used for subsequent junctional prototype testing; however, this approach does not reach full occlusion, only visible loss of arterial lumen (Fig. 5A). Additional time to reach occlusion was measured using the junctional ultrasound guidance and occlusion (JUGO) phantom by recording the time from reaching an AI decision by the occlusion AI to when the actuator reached full occlusion, defined as 90% reduction in flow rate. Overall, the AI classification identified occlusion after approximately 10s while an additional 6.8 ± 1.1s was required to reach full occlusion (Fig. 5B). As such, the prototypes were programmed to continue actuation for this additional duration to ensure hemorrhage control.

### Real-time testing AI-driven junctional tourniquet prototypes

**Fig. 6:**
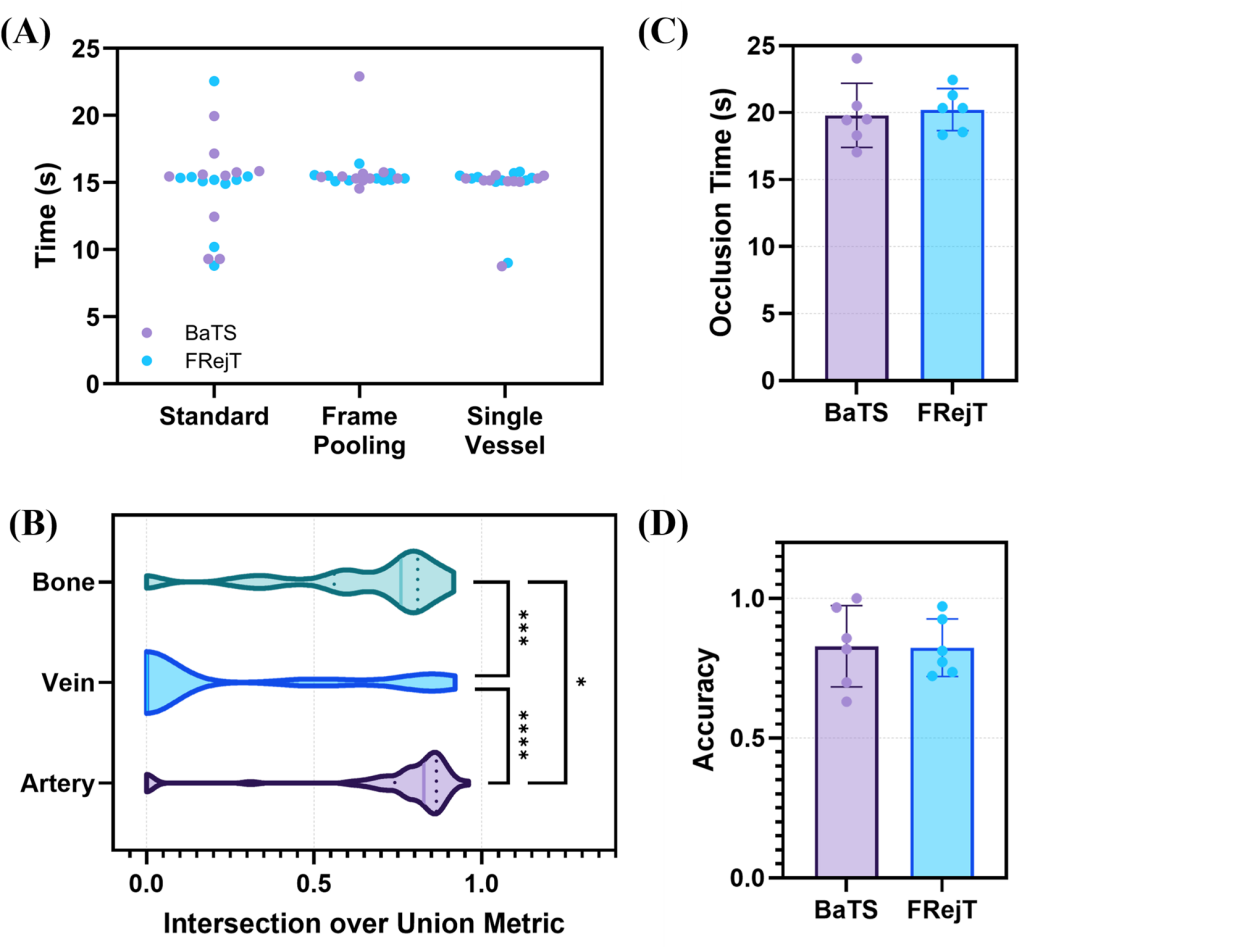
Proof-of-concept testing results for real-time prototypes. (**A**) Comparison of performance between normal, frame pooling, and single vessel object detection configurations across *n* = 10 replicates real-time runs for BaTS and FRejT. Differences between guidance timings were not significant as determined by Kruskal-Wallis, post hoc Dunn’s test. (**B**) IOU scores for each captured frame for bone, vein, and artery features. Significance differences between groups (* p-value < 0.05; *** p-value < 0.001; **** p-value < 0.0001) were determined by Kruskal-Wallis test, post hoc Dunn’s test. Overview of (**C**) time to occlusion and (**D**) occlusion accuracies for BaTS and FRejT. Differences between groups were not significant as determined by unpaired T-Test.

For final proof-of-concept testing, prototype designs were integrated with AI models using a single board computer for performing model inferences and driving mechanical actuation in real-time. Testing was performed using the femoral JUGO phantom for both the BaTS and FRejT designs. Initial prototype testing was hindered because the object detection logic of the anatomical guidance model did not recognize the necessary landmarks to trigger occlusion actuation. This was debugged by testing three different guidance AI model integrations configurations – (i) wherein the model was used on each frame to identify each anatomical landmark, or (ii) only a majority of the pooled frames had to have positive identifications, or (iii) a vessel (artery or vein) and bone were sufficient to start occlusion actuation. The number of frames, and, thus, the time required to start occlusion was measured for the three guidance model configuration in 20 replicate runs split evenly between its use with FRejT and BaTS. Overall, the three guidance configurations had similar times to trigger actuation (Fig. 6A), however, image pooling and vessel/bone protocols had lower coefficients of variation at 10.9% and 13.6%, respectively compared to 21.1% for the standard method. Real-time IOUs showed that the artery and bone were both significantly more often detected by the AI model than vein (Artery vs. Vein p-value < 0.0001; Bone vs. Vein p-value = 0.0004; Fig. 6B), thus, the single vessel/bone protocol was selected as the best guidance method for future testing.

During the second round of prototype testing using the single vessel/bone guidance protocol, time to reach occlusion was quicker, at approximately 20s for both prototype designs (BaTS = 20.2 ± 1.6s; FRejT = 19.8 ± 2.4s; Fig. 6C). Classification AI model accuracy during occlusion was similar with BaTS and FRejT at 0.829 ± 0.145 and 0.824 ± 0.103, respectively (Fig. 6D). In summary, the prototypes were successfully integrated with AI models for guidance and occlusion tracking and were quick to maintain occlusion, highlighting the potential for automated junctional tourniquet technology.

## Discussion

Junctional hemorrhage control remains a mostly unsolved problem for prehospital civilian and military medicine. It is difficult to properly align current tourniquet and maintain sufficient occlusion during patient transport. While improved personnel training can improve junctional hemorrhage management, there remains an unmet need to simplify and automate junctional tourniquet devices through integration of engineering improvements. The prototype devices paired with AI models, as demonstrated in this proof-of-concept study, have the potential to improve hemorrhage control for one of the leading causes of traumatic preventable death.

The two junctional tourniquet prototype designs – FRejT and BaTS – were developed in response to the limitations identified with existing junctional tourniquet technology. Each design reflects a distinct approach to meeting various design criteria such as device stability, adaptability, and integration with an AI guidance and occlusion framework. Both designs are centered around a portable ultrasound transducer (L7 HD3 Linear Scanner, Clarius, Vancouver, BC, Canada). The wireless form factor of the US probe and its relatively lightweight packaging allows the junctional tourniquets to be easily deployed. Overall performance across design metrics is summarized in Table 1 for each design. The highest priority design criteria were given to time to occlusion, stability, and overall occlusion effectiveness—factors most critical to the survival of the patient in emergency scenarios. Other factors, such as force applied, were not investigated in this study and will be measured in future studies.

**Table 1:** Summary of design criteria and performance for each junctional tourniquet prototype design.

Priority	Criteria	BaTS	FRejT
High	Total time to reach occlusion during timing characterization	Assembly Time: 17.9s	Assembly Time: 18.7s
		Securement Time: 11.7s	Securement Time: 5.72s
		Occlusion Time: 48.4s	Occlusion Time: 24.0s
		Overall Time: 78.0s	Overall Time: 48.4s
	Stability of the tourniquet at the anatomical sites	2 device slips during timing characterization	0 device slips during timing characterization
	Effectiveness of occlusion by the tourniquet	Successfully achieved full occlusion (flow < 10%) each run	Successfully achieved full occlusion (flow < 10%) each run
Normal	Ultrasound compatibility of the tourniquet	Pass	Pass
	Versatility and ease of use	Worked at all anatomical sites comparable to commercial options	Worked at all anatomical sites comparable to commercial options
	Durability	Straps improve durability by having fewer rigid parts	More rigid parts have increased degradation
	Portability	Weight = 2.39 kg; Current dependency on rail system makes it less portable	Weight = 2.77 kg; Similar in footprint to commercial options

Within these criteria, FRejT outperformed BaTS by achieving the fastest assembly to occlusion time at 48.4s vs. 78.0s, demonstrated consistent stability, with no tests runs slipping or failing to reach occlusion. Two BaTS trial runs slipped during initial placement due to the higher skill level required to position the straps of the prototype compared to the rigid frame of FRejT. BaTS and FRejT had more comparable assembly and securement times, but a larger difference was evident during occlusion with BaTS requiring double the time of FRejT. However, this difference in occlusion time was resolved with the integration of the AI models, as occlusion time was nearly identical for each prototype during RT prototype testing. This highlights the benefits of the AI models which were missing during initial characterization and dramatically improved reliability of the BaTS device when integrated. Secondary design criteria included versatility/ease of use, durability, and portability—important attributes that, while not directly tied to life-saving function, still impact real-world deployment.

Focusing on the FRejT prototype, the system focused on structural rigidity and precise probe movement by incorporating a three-component design to expedite the setup process. The use of aluminum framing and modular t-slotted components allowed for repeatable, adjustable positioning of the ultrasound probe. The disk interface between the actuator and the probe holder allowed for easy insertion and removal, as well as rotational adjustment of the US probe. The placement of the actuator and probe holder allows for more uniform mechanical force distribution, reducing the possibility of slippage. Although the FRejT system proved to be effective, there are some limitations to the current design. The three components used to assemble the system are fully separate from each other, which could lead to incorrect assembly under stressful or hostile conditions leading to device failure. Furthermore, the device needs to be placed beneath the patient during assembly, which requires the operator to lift or move a trauma patient which may be impractical. Additionally, the design’s actuation mechanism only allows for pressure to be applied in the z-axis. This makes positioning the US probe difficult in the subclavian scan site, increasing the possibility of device slippage.

As for the BaTS prototype, its design was focused on adaptability to a variety of anatomical contours by replacing a rigid base with a strap-based stabilization system. The adjustable straps allowed the precise positioning of the US probe at different occlusion sites while maintaining perpendicular contact with the skin’s surface, which is particularly useful in regions with pronounced curvature such as the subclavian occlusion site. The straps are manually tightened, reducing the workload on the actuation mechanism. Additionally, aside from an attachment railing that is pre-installed on a table or gurney, BaTS is constructed as a single component, simplifying the assembly process. While this design was constructed to be versatile, the design requires the installation or adaptation of a gurney or table to incorporate the railing system. During testing, device performance was highly dependent on the user’s ability to secure the device perpendicular to the skin, otherwise the device would slip when the actuator began extending. Additionally, the device can be cumbersome to be deployed by a single user due to its large size and straps getting tangled. Further work will focus on enhanced ruggedization of the design to improve portability, deployment and durability of the prototyped device and address the discussed limitations related to initial setup and securement of the device to the subject. Additionally, both BaTS and FRejT could see improved ease of deployment by expanding the current 1-dimensional linear actuator driven automated movement to a 3-dimensional algorithm that procedurally locates the hemorrhaging junctional site before applying pressure and occluding the site with the prototyped device.

While both designs had their advantages and disadvantages, each device successfully integrated the AI models to automate junctional hemorrhage control. The AI models discussed in this study were developed for two purposes – (i) identifying the anatomy responsible for inducing junctional hemorrhage and (ii) determining the occlusion state of the underlying vessels. The anatomical guidance object detection AI model was successfully trained and implemented with IOU performance metric scoring higher than a 0.5 threshold on blind holdout data and real-time testing, yet was influenced by US image quality and anatomical feature position during image capture. US images where the objects in view were very clear had higher IOU scores compared to lower performance when the US images were captured at different probe angles, tissue compression levels, and other slight capture modifications. In fact, some test runs observed venous IOU scores drop below 0.23 in real-time use resulting in the need to base guidance decisions on detection of a single vessel and bone instead of artery, vein, and bone. This improved real-time performance consistency but is not optimal for future implementations of this technology. Additionally, the recall and IOU scores for the bone were noticeably lower than the other two classes. This could be due to the artificial nature of the bone in the tissue phantom and its shape changing while the US probe changed angle and orientation in a way that is not presented in the training data for the model. In future work, greater variance in training data is required to be able to identify this different view of the anatomy. In addition, alternative state-of-the-art object detection model architectures such as Detectron2^37^, EfficientDet^38^, and YOLOv12^39^ or the integration of semantic segmentation models for more precise anatomical labeling^40–42^ may improve performance.

Different approaches were evaluated for occlusion detection using classification AI models with the goal of identifying a consistent detection point without the need for color Doppler^27^, as it is not always practical for portable US systems and the phantom testing setup used was not Doppler compliant. The initial approach taken was to train a model to identify the quantitative flow reduction levels, but this ultimately failed to accurately identify classes past baseline as evidenced by Fig. 3. This could be due to class imbalances across the quantitative flow levels. Another challenge with the dataset was that US images at each quantitative flow level were similar, likely responsible for the poor performance. Further, flow rate reductions can be a lagging indicator of occlusion due to the compliance of the tubing in the tissue phantom models used. Therefore, rather than relying on the precise flow rates to determine how to train the occlusion model, a qualitative approach to determine occlusion was the most consistent solution for this application. However, the time to reach true occlusion from when our classification model detects occlusion is heavily dependent on our testing platform, making it unlikely to transfer to a different subject or setting. While training a classification model to detect the point of occlusion where arterial flow drops below 10% of full flow would be ideal, the current system still provides a benefit where the time of actuation between the model detecting the closed lumen and absolute arterial flow stop is more consistent than the alternative where the motor would extend against the site for a fixed amount of time upon deployment. The latter could face discrepancies in efficacy as different positions or angles of the prototype against the site or differences in subject physiological makeup could yield different times to total occlusion, while such inconsistencies are less likely in the AI driven method as the fixed window of actuation is much smaller. Nevertheless, in future iterations of the AI integrated prototypes, actuation force must be measured to ensure the devices are not exerting excessive force on the underlying tissue. A more robust dataset, with higher subject variability, would provide additional training resources for the AI models and opportunity for refining the method of detecting occlusion to achieve a complete AI driven device.

Overall, this study and AI model training were conducted on tissue-mimicking phantoms. While these provided the necessary platform for the proof-of-concept design and evaluation of the fully integrated junctional tourniquet prototypes further evaluation on live tissue is necessary for full product development. Tissue phantoms do not provide the subject variability or tissue complexity that live tissue does, and therefore AI models trained on these platforms could never be deployment ready. However, using tissue phantoms for initial AI training can provide a basis for later transfer learning models with live tissue data, therefore reducing the number of humans and/or animals used for research purposes^43^. Other limitations of the tissue phantoms used for this testing included the need for multiple materials, one used for timing characterization since it was able to withstand repeated compression but had minimal ultrasound compliance, while the ultrasound compliant material provided a more robust test platform but many times teared during testing. For the aortic phantom, a base layer was added for ease of testing that provided a flat posterior so the phantom would be stable for bench-top testing. Another aspect that can be improved is capturing prototype use with more than one end-user. During this testing, a single user with prior tourniquet use experience in trauma settings performed the timing characterization testing for all tourniquets. With future, more advanced iterations of the devices and AI models, human usability studies will be conducted to capture end-user experience and device performance in a more adequate environment.

In conclusion, improved solutions for achieving junctional hemorrhage control are crucial for civilian and military medicine. The prototypes presented here demonstrate how ultrasound imaging coupled with AI models can be used to enhance junctional hemorrhage control. However, this study was proof-of-concept in nature, and, thus, further refinement and testing is needed to launch these prototypes into a deployable, production format. Future development will focus on improving design features and user guidance from the AI models, as well as testing occlusion in more relevant models such as live animals and human cadavers. Ongoing advancements for this technology will enhance the use and accuracy of junctional tourniquets, especially when used for prolonged periods of time.

## Materials and methods

This section first details the design and fabrication process for the two junctional tourniquet prototypes, followed by a description of the various test platforms used. Specifically, three different test platforms were used – (i) a commercial tissue mimicking phantom of the human torso for evaluating tourniquet placement times, (ii) custom tissue-mimicking phantoms and flow loops of the subclavian, aorta, and femoral junctional sites suitable for timing characterization of junctional tourniquets to vessel occlusion, and (iii) an ultrasound-compatible junctional ultrasound guidance and occlusion (JUGO) phantom suitable for training AI models and testing full prototype performance in real-time performance. After introducing each tissue phantom, the development and testing of AI models used to power device functionality is described for both the guidance and occlusion tracking AI models, along with how performance for these models was assessed. Finally, real-time testing of the AI-driven junctional tourniquet prototypes is detailed along with performance characterization.

### Design and fabrication of US-enabled junctional tourniquets

The design criteria for the automated junctional tourniquet prototypes were categorized based on priority to guide development and evaluation. High-priority considerations included minimizing the total time required to achieve occlusion, ensuring stability of the tourniquet at the targeted location without slippage, and occlusion effectiveness. Normal priority criteria ensured the compatibility of a wireless ultrasound transducer, ease of operation for the user, and portability. The transducer used was a portable linear array scanner (L7 HD3 Linear Scanner, Clarius, Vancouver, BC, Canada) with 4 to 13 MHz frequency, weighing 288 g, and measuring 147 × 76 × 32 mm. Both prototype designs were based on this transducer as linear arrays are commonly used for visualizing femoral vessels, and it was a portable linear probe that allows backend communication for AI-based image interpretation. Identified low-priority criteria at this stage in the design process were device reusability, battery life, and cost affordability of the tourniquet prototypes, but were not taken into consideration at this stage. However, low-priority criteria were not considered for the current prototype iterations designed in this work. With these design criteria in mind, two different tourniquet prototypes were developed as detailed below.

#### Frame reinforced junctional tourniquet

**Fig. 7:**
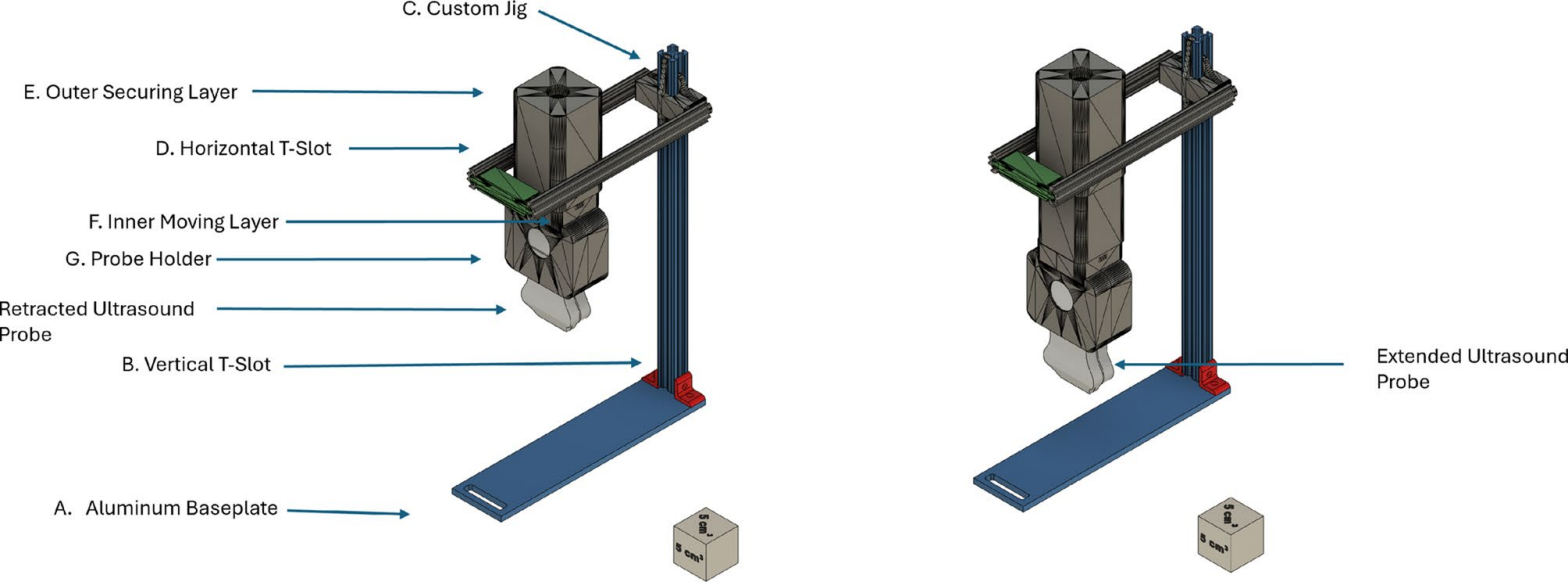
Design of the Frame Reinforced Junctional Tourniquet (FRejT). A computer-aided design overview of the FRejT prototype is shown along with the labels for the (**A**) aluminum base plate, (**B**) vertical t-slot extrusion, (**C**) 3D-printed jig for attachment to (**D**) horizontal t-slot extrusions, (E – G) main housing of the US probe comprised of (**E**) outer securing layer, (**F**) inner moving layer, and (**G**) probe holder.

The prototyping stage of the Frame Reinforced Junctional Tourniquet (FRejT) followed a similar frame-based tourniquet structural design to the Combat ReadyClamp (CRoC). This design criteria aimed to preserve its portability and convenient design characteristics, while introducing automation to enhance functionality. A diagram of the final version of FRejT is shown in Fig. 7, as a drawing from the computer aided design (CAD) software (Autodesk, San Francisco, CA, USA). In summary, the frame structure is coupled to a 3D-printed housing for the portable ultrasound probe and linear actuator to enable automated junctional occlusion actuation driven by AI models.

Initially, the need for stability of the system led to the machining of an aluminum base plate (Fig. 7, Label A), designed to secure the device using a Velcro strap. To ensure flexibility in the positioning of the probe, T-slotted aluminum extrusions (McMaster-Carr, Elmhurst, IL, USA) were utilized, allowing free movement of the system. A vertical t-slot (Fig. 7, Label B) secured by L-brackets enabled a 90° pivoting range relative to the base, and a custom jig (Fig. 7, Label C) was 3D printed to attach horizontal t-slots (Fig. 7, Label D), providing dual-axis movement. The original prototype had the main body secured to the horizontal t-slot on only one side, which resulted in uneven pressure distribution and frequent US probe slippage from its magnetically secured housing. Incremental changes to the system were insufficient to resolve these issues until a substantial design revision was implemented. This major change involved splitting a T-slotted frame and securing each half to the custom jig, centering the main body and distributing force evenly.

The redesigned main body is composed of 3 parts: the outer securing layer, the inner moving layer, and the probe holder: Figs. 7 labels E, F, and G respectively. The outer securing layer slides onto the two-horizontal t-slots and houses the NEMA-23 (Haydon Kerk, Waterbury, CT, USA) linear actuator that drives the inner moving layer. The end of the inner moving layer has a 5 cm thick disk, which the probe holder clamps onto. This disk allows the user to rotate the probe holder and modify the angle at which US scans are being evaluated, which can help identify the correct positioning and occlusion. The probe holder itself was modified from a front-back sandwich design to a left-right orientation, drastically enhancing the stability of the probe holder under pressure. Lastly, a Velcro strap attaches from the base plate to the end piece allowing the user to securely tighten the entire assembly, further preventing unwanted movement during use of the device.

#### Base and tightening straps

**Fig. 8:**
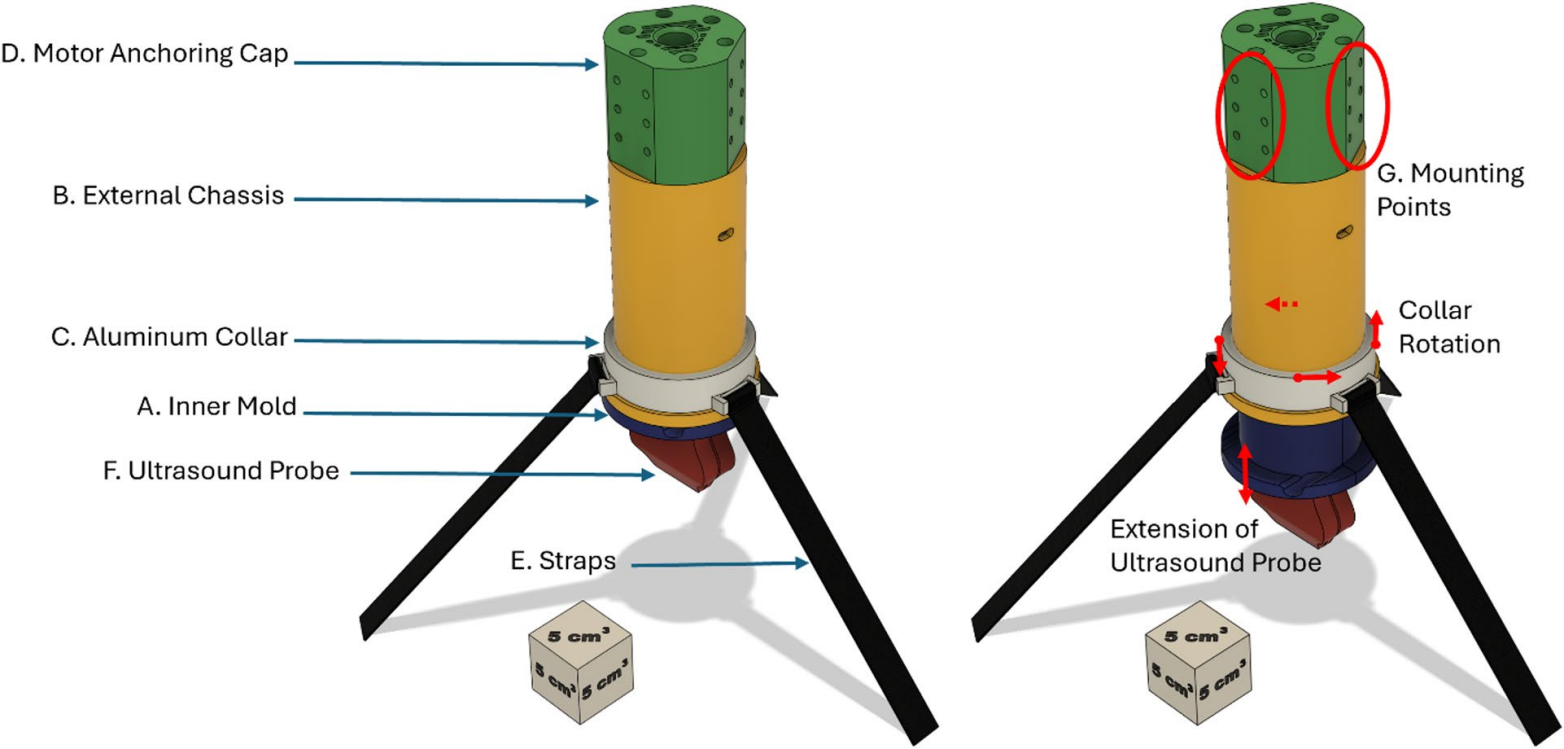
Design of the Base and Tightening Straps Junctional Tourniquet Prototype. A computer-aided design overview of the BaTS prototype is shown along with the labels for the (**A**) inner mold, (**B**) external chassis, (**C**) aluminum collar, (**D**) motor anchoring cap, (**E**) straps, (**F**) ultrasound probe, and (**G**) mounting points for handle or electronic attachment. The different views of the prototype show the extension of the US probe by linear actuation during occlusion and collar rotation functionality.

The main goal of the Base and Tightening Straps (BaTS) junctional tourniquet design was to develop a device suitable for adequate tissue contact for occlusion regardless of the curvature of the underlying site. To do so, the design deviated from FRejT prototype by not relying on a base that the probe could stabilize upon but instead utilizes three straps attached to a spinning collar that is affixed to the device. The straps, which are anchored to the table or gurney after initial occlusion, allow BaTS to be held on any occlusion site while maintaining the optimal angle. The straps can be adjusted and tightened to different lengths to keep the device normal to the surface of the patient (Fig. 8).

The BaTS prototype consists of an inner mold (Fig. 8, Label A), an external chassis (Fig. 7, Label B), an aluminum spinning collar (Fig. 8, Label C), a motor anchoring cap (Fig. 7, Label D), and nylon straps (Fig. 8, Label E). The BaTS junctional tourniquet was designed using Autodesk CAD suite and was primarily fabricated with 3D extruded parts made of PLA. The inner mold holds the Clarius US probe (Fig. 8, Label F) so that the transducer becomes the point of contact to the occlusion site. The inner mold is then attached to the base of a NEMA-23 linear actuator whose end is bolted to the top of the outer chassis with a single ¼” − 20 nut. This allows the inner mold to telescope in and out of the outer chassis as needed to achieve occlusion. The aluminum collar is then slipped over the outer chassis and the straps attached to the collar at equidistant anchor points. The spinning collar design is important as it allows the operator to intuitively determine how BaTS is supposed to be anchored to the table or gurney and tighten the straps at different lengths, allowing the probe to be optimally angled for maximum occlusion and stability. The motor anchoring cap was designed to accommodate various handle configurations and single-board computer mounting (Fig. 8, Label G) in future design iterations.

### Anatomical phantoms for junctional tourniquet placement

#### Casting and molding of perfused tissue phantoms

For troubleshooting and testing junctional tourniquet prototype time to assembly, placement, and occlusion we used a combination of two tissue phantoms: a commercial FAST full torso phantom with head and hip/upper-thigh region^44^ and a custom tissue-mimicking phantoms made of silicone elastomers that allowed perfusion. Assembly of the prototypes and placement at the correct occlusion site were captured on the commercial phantom. Time to fully occlude vessels once positioned properly was assessed in the perfused custom phantoms for each region: subclavian, aortic and femoral.

Each custom phantom was molded from a commercial mannequin (Amazon, Seattle, WA, USA). The mannequin was broken into three pieces that included chest-to-head, mid-torso, and hip-to-upper thigh regions for each occlusion site respectively (subclavian, aortic, and femoral). The plastic cutout was vacuum formed with a Formech450DT (Formech Inc., Middleton, WI, USA) with acrylic sheets (McMaster-Carr, Elmhust, IL, USA). Circular cutouts were made in the acrylic molds at the distal and proximal ends of the occlusion site to allow for a vessel placeholder to be placed while curing. For the venous channels, the tubing was compressed into an elliptical shape to mimic the shape of a hypovolemic vein. For all vessels, the tubing was shaped to its anatomical path as closely as possible and placed inside the mold. The vessel cutouts were sealed using SilPoxy (Smooth-On Inc., Macungie, PA, USA) and the mold was sprayed with silicone mold release (CRC Industries Inc., Warminster, PA, USA). For each region, the custom tissue phantoms had specific 3D printed bones and curing methods described below.

##### Subclavian region

Using an open access repository of 3D body parts models^45^, we modified a CAD of the bone assembly using the first to third vertebra, the left and right first to third ribs, clavicles, scapulas and cut humerus. These were attached to the sternum and manubrium by slightly overlapping the bones in SolidWorks (Dassault Systèmes, Vélizy-Villacoublay, France). Due to the 3D printer size restraints, the bone CAD was split through the sagittal plane. Both halves were printed using PLA (polylactic acid) filament in a Bambu Labs X1C printer (Bambu Lab, Shenzhen, China), and then pasted with Gorilla Glue epoxy (Cincinnati, OH, USA).

For mold assembly, the bones were suspended to avoid contact with the walls and surface at the approximate anatomical location, relative to the mold, and the copper tubing (McMaster-Carr, Elmhurst, IL, USA) vessel place holders were accommodated for subclavian occlusion. Once vessels and bones were secured, about 6 L of EcoFlex Gel2 (Smooth-On Inc., Macungie, PA, USA) were poured into the mold and degassed in a vacuum chamber at − 25 inHg (~ −84.7 kPa) for 30 min before curing for 2 h. Once cured, the copper tubing was removed, and the phantom was released from the mold. For perfusion, ¼ inch (6.35 mm) Penrose drainage tubing was inserted into the artery and vein channels and attached to a flow loop setup for occlusion testing described later.

##### Aortic region

The phantom backbone was simulated from a 3D printed spine. A CAD was created in SolidWorks Software using the vertebrae, first and second lumbar vertebra and 4th to 12th thoracic vertebra, using the same open access repository^45^. The individual vertebrae were slightly overlapped together to create a solid spine and was 3D printed using PLA in a Raise 3D Pro printer (Irvine, CA, USA). Due to the size of the inferior vena cava and abdominal aorta, vessel channels were also 3D printed using 150 mm long 3D printed PLA cylinders. To model vessel channels, a cylinder (15 mm diameter) was used for the artery and an elliptical cylinder (13 mm height and 22 mm width) for the vein.

The aortic phantom also used approximately 6 L of EcoFlex Gel 2 and had the same degassing and curing procedure. In addition, this tissue phantom required a 4 L layer of EcoFlex 00–20 (Smooth-On Inc., Macungie, PA, USA) to provide a rigid base while scanning, that was directly poured and cured to the back of the phantom. Once all layers were cured, the vessel channels were removed, and the phantom was released from the mold. Penrose drainage tubing – ¾ inch (19.1 mm) diameter for the vein and ½ inch (12.7 mm) for the artery – was placed inside the vessel channels and attached to a flow loop setup for subsequent testing.

##### Femoral region

The bone structure used for the femoral phantom was assembled using the left hip and femur from an anatomical skeleton model (Amazon, Seattle, WA, USA), and pasted with RTV Silicone adhesive (Pro Seal, Pacer Technology, Rancho Cucamonga, CA, USA). The distal end of the femur was cut to ease manipulation and fit the mold. Once the bones and copper tubing (as vessel placeholders) were placed in the mold, two layers of approximately 2 L each of EcoFlex Gel2 were poured and degassed in a vacuum chamber at −25 inHg (~ −84.7 kPa) for 30 min. Once cured, the copper tubing was removed, and ¼ inch (6.35 mm) Penrose tubing was placed in the channels and attached to a flow loop setup for occlusion testing described in subsequent sections.

#### Timing characterization of commercial and prototype junctional tourniquets

Timing characterization was conducted in order to compare the practical use of the prototype devices to commercial devices. The commercial tourniquets used were CRoC, SJT, and AAJT. Timing was characterized in three categories – (i) assembly, (ii) securement, and (iii) occlusion time. As the assembly and securement timing measurements benefitted from full torso anatomy, assembly and securement timings were collected with a FAST Exam Ultrasound Training Model (Blue Phantom, Redmond, WA, USA)^44^. Testing consisted of a single user, with real-world experience in using tourniquets to assemble and secure each tourniquet to each of three anatomical locations – subclavian, aortic, and femoral regions. For each tourniquet, assembly timing was measured from a fully disassembled state to an operational state, while securement timing was measured from the end of assembly to proper placement over the junctional site. The assembly and securement processes were timed for three iterative runs (*n* = 3) for each tourniquet and each anatomical location, by a single operator for all tourniquets and all sites. Due to some commercial tourniquets not being compatible with all anatomical sites, some tourniquets were not applied to every phantom; AAJT was used only for the aortic region, SJT was used in the femoral and subclavian regions, and CRoC was used at all three anatomical sites. Both prototypes, BaTS and FRejT, were designed for and used at all sites.

For occlusion timing of each junctional tourniquet, the custom tissue phantoms were perfused in flow loops consisting of a peristaltic pump (Masterflex, Vernon Hills, Illinois, USA) circulating water, attached to the proximal side of the arterial tubing, with a bypass for post-occlusion flow diversion, and pressure sensors to monitor flow loop state using a Dräger monitoring system (Dräger, Inc., Lübeck, Germany). For the aortic region with larger tubing, high flow rates were required (300 mL/min), and a double tubing peristaltic pump head was used (Masterflex, Vernon Hills, Illinois, USA); a single peristaltic pump head was adequate for the lower flow rates of the subclavian and femoral regions (flowrates averaging 110 mL/min). The distal tubing end was open to a fluid reservoir to capture outflow and simulate a bleed after fluid passing through a flow sensor (FD-X, Keyence, Osaka, Japan) for occlusion confirmation. The mean pressure in the flow loop was held around 75 mmHg by adjusting pump flow rates and the overall fluidic resistance in the bypass and bleed flow paths using various gauge blunt needle connectors (McMaster-Carr, Elmhurst, IL, USA). All data, pressure and flow sensor outputs were collected via a PowerLabs 16/35 (ADInstruments, Sydney, Australia) data acquisition system, and processed using the LabChart 8 (ADInstruments, Sydney, Australia) software. For the venous side of each flow loop, the Penrose tubing was filled with water and left open to air at hydrostatic pressure to maintain proper vessel volume but not necessarily at a specific pressure.

Recording occlusion timing began after pre-assembling each device and positioning at the occlusion site, without applying pressure. Testing protocol started with an ongoing simulated bleed, recording a 30s baseline measurement of flow rate. Then, each tourniquet was manually applied, increasing vessel occlusion at the site while monitoring flow rate reduction to confirm occlusion. Full occlusion was verified in two ways: by visual confirmation of drip stop and flow sensor showing a reduction below 10% of baseline flow. Upon achieving occlusion, it was maintained for one minute taking note of signs of slippage or any sudden increase in flow rate. The test concluded with the release of each tourniquet from the occlusion site followed by an additional 30s baseline to confirm the flow loop returned to normal values. Occlusion timing was measured from the start of tourniquet actuation (activating motors or filling air bladders) to the first occlusion criteria that was met (visual drip stop or flow sensor) at each anatomical site. Timings for each stage were annotated in the data acquisition software for three replicate runs, enabling comparative analysis of tourniquet performance.

### AI Model development and training

#### Functional femoral tissue-mimicking phantom

##### Making of phantom for ultrasound guidance and occlusion

In addition to characterizing the timings of the junctional tourniquet prototypes, this research also looked at the ability to integrate AI models for interpretation of the ultrasound scans while operating the BaTS or FRejT prototypes. This required testing with a tissue phantom that was both compatible with ultrasound imaging and able to withstand mechanical compression. Only the femoral compression site was pursued for this phase, and a phantom was made using DIY Gel Peach (Humimic Medical, Greenville, SC, USA) for ultrasound guidance compatibility and occlusion; hence termed the Junctional Ultrasound Guidance and Occlusion (JUGO) phantom. The material acoustic and image properties of the DIY Gel dyed with peach coloring are 832.97 Kg/m^3^ as the density, 1431.7 m/s as the speed of sound and an acoustic impedance of 1.19e6 Rayls. This material is synthetic gelatin made explicitly for manufacturing life-like tissue phantoms and is shelf-stable and reusable. A slightly different casting process was used to fabricate the mold for this phantom, since it must be high-temperature resistant for the melted DIY Gel material. The mold for JUGO was made out of PC (polycarbonate) using the same vacuum former device over a Clear Ballistics Gel 20% (CBG20, Clear Ballistics, Greenville, SC, USA) tissue phantom of the region. The CBG20 phantom was only used as basis for the mold since it was not fitted with anatomical features and during preliminary testing did not withstand the mechanical compression needed for junctional tourniquet testing.

For the anatomical feature in the JUGO phantom, a similar approach was used with copper tubing to create vessel channels after tubing was engineered to follow venous and arterial paths and shape. The hip and femur bones were 3D-printed from CAD files of human anatomical structure^45^ using High Temp V2 resin (FormLabs, Somerville, MA, USA) and pasted together. Once the bones were fixed, they were placed in the mold with the vessels and coated with silicone release spray for ease of removal. Approximately 3 L of DIY Gel Peach were poured after melting at 130 °C in a laboratory oven (Thermo Scientific, Waltham, MA, USA) for the JUGO phantom. This was allowed to solidify at room temperature for at least four hours. No degassing was necessary as trapped air was released during the initial melting process, and the phantom was poured carefully to avoid air introduction. Once fully cured, the copper tubing was removed, and the JUGO phantom was released from the mold ready for data collection and testing.

##### Flow-loop assembly and data collection

A similar flow-loop, as described in the timing characterization section, was used with the JUGO phantom. Briefly, perfusion of the arterial side was maintained using a peristaltic pump and mean pressure was maintained around 75 mmHg with the presence of an open bleed, while the vein was maintained under hydrostatic pressure. Once the flow loop was set up, four types of US scans were collected with the Clarius US system to train AI models with 10s video clips captured for each in the JUGO phantom: probe sliding across the inguinal creases in lateral motion, probe tilting with vessels centered ± 45º, probe rotating ± 180º, and probe compressing levels to different degrees of occlusion (50%, 60%, 70%, 80%, 90%, and 100% flow reduction). US feed was collected using a video capture card connected to a computer and recorded with the LabChart 8 software along with the flow sensor data and pressure readings. At least three 10s clips were captured for each motion, in six different JUGO phantom pours to introduce subject variability in the training dataset for the guidance model and occlusion models. The guidance or object detection models used data from all video types while the occlusion, classification model only used data from the compression videos. An additional JUGO phantom was later poured to capture videos of each type and serve as a blind subject AI model for performance benchmarking.

For the compression videos, the scanning protocol for each occlusion level was automated by attaching the US probe to a linear actuator held above the femoral JUGO phantom. A 30s baseline was collected at no compression, then the actuator was manually controlled to stepwise compress the vessels to the different degrees of occlusion as quantified by flow rate sensor until reaching the desired occlusion percentage. This scanning protocol was collected for 3 iterative runs across the six initial JUGO Phantom pours, for each of six levels of flow occlusion (50%, 60%, 70%, 80%, 90%, and 100%). Occlusion was then held for 10s followed by actuator retraction until baseline flow resumed.

#### Object detection – guidance models

All US frames were extracted from the videos collected from the JUGO phantom and annotated for training and scoring object detection models for guidance to the correct anatomical location. Training data was further split at approximately a 2:1 ratio between training and validation, resulting in four of six phantoms used for training while the remaining two phantoms were used for validation during training. The guidance AI model was setup to track three anatomical features essential for junctional tourniquet function – the bone, artery and vein. Each of these features was manually labeled with bounding boxes using the MATLAB computer vision toolbox (MathWorks, Natick, MA, USA). Ground truth data used for training had 30,924 annotated frames (42 ultrasound videos), while validation data had 11,706 annotated frames (24 ultrasound videos). The videos used for blind testing were annotated using the same process and totaled 2,548 frames (4 ultrasound videos).

The object detection model trained was YOLOv8n, due to its superior inferencing speed compared to other object detection AI model architectures with a limited trade-off in accuracy, making it an ideal fit with our application requiring US data interpretation in real-time. Training was conducted over 100 epochs and utilized default YOLO training parameters and default random image augmentations (hue, saturation, and brightness random image adjustments; horizontal and vertical image translations; random image scaling; left-right image flipping; combination of 4 images into a mosaic), and pretrained weights^37,46^. The computer hardware setup that the training was conducted on had the following specifications: an NVIDIA Geforce RTX 4090 24GB GDDR6 GPU, a 12th gen Intel Core i9-12900 K CPU, 128GB RAM with an Ubuntu 22.04 operating system. Performance was based on three metrics – precision, recall, and intersection over union (IOU)^47^. Each metric was calculated per video captured for the blind testing femoral phantom to see at which view the object detection model performed best at and where it struggled. Overall performance was then calculated by taking the average of these metrics across all four videos.

#### Classification – occlusion models

The occlusion classification model utilized the same YOLO architecture that was used to develop the object detection model described above but utilized the classification YOLOv8n-cls pretrained weights. US frame occlusion data was extracted from the femoral JUGO phantoms for training, validation and testing, similar to the object detection model. Summary of training and validation ultrasound dataset sizes for each model are shown in Table 2; all occlusion AI models were tested on a single blind video of 754 frames. It is important to note that for the Multi-Class and 3-Class models, validation was not performed on blind phantom legs, resulting in both models being overfitted to the data represented in training. The training was conducted using default training parameters and image augmentations, using the same hardware described above.

**Table 2:** Data summary for training classification models.

	Multi-class		3-class		2-class		Qualitative	
	Video	Frame	Video	Frame	Video	Frame	Video	Frame
Training	16	7,165	16	7,165	12	6,533	12	8,412
Validation	7	552	7	552	7	3,210	7	5,707

Different training approaches were attempted for the classification model where each approach represented a different way the vessels in view were classified as “occluded”. A total of four different methods were developed where each iteration simplified the classification task being conducted. The first model developed was intended to be able to classify all US frames under 50% occlusion as “baseline” before detecting each 10% occluded step up from 50% to 100% as a separate class, yielding 7 total classes. The second classification model was a 3-class model that was trained to detect “baseline” US images where no pressure was being applied to the site, “in-progress” frames that were comprised of US images from beginning of compression to 80% flow reduction, and “occluded” images where the vessels had a 90%−100% flow reduction. The third model was a 2-class model where data that was captured under 50% flow reduction was classified as a “baseline” image while all images above this flow reduction were classified as “occluded”.

Lastly, for the fourth model, we moved away from the quantitative splitting of US frames into classes and instead used a qualitative approach. In this approach, if any vessel lumen was visible in the artery of the JUGO phantom, the US frame was classified as a “baseline” image; once the lumen was not visible (i.e. vessel fully collapsed) the frames were assigned to the “occluded” category. Qualitative image curation as well as bounding box generation for the object detection model was conducted by the study research team. For practical application of this fourth model iteration, the time to achieve flow reduction above 90% after lack of lumen visibility was experimentally determined as approximately 6s of actuation. For this determination, the same linear actuator as the one integrated into the junctional tourniquet prototypes was used. Performance of all occlusion models was based on blind testing US data and the accuracy of categorical prediction for each model setup.

### Real-time testing of junctional tourniquet prototypes

AI guided real-time testing was conducted by driving both the FRejT and BaTS prototypes’ functionality through inferences run by both trained AI models. These tests integrated real-time inferencing of AI models controlling the linear stepper motors on the FRejT and BaTS prototypes via a single board computer, the Jetson Nano (NVIDIA, Santa Clara, CA, USA). The Jetson Nano controlled the motors by sending signals to an A4988 Motor Driver (Pololu, Las Vegas, NV, USA) per manufacturer instructions, which would then extend the motor to occlusion or retract it to reset positioning. Using the femoral JUGO phantom with the flow loop setup, these tests began with a 30s baseline measurement, followed by an assembly and positioning of the tourniquet. After the tourniquet was in position, the AI models interpreted streamed images from the Clarius US probe, where the object detection model determined if the relevant anatomical landmarks were in view before initiating actuation.

Next, the qualitative occlusion AI model provided inferences about occlusion status wherein if occlusion was detected in a single streamed frame, the model confirmed this result by evaluating over the next five frames. If four out of five were evaluated as “occluded”, the motor actuates for an additional 6s to reach “true occlusion” of the artery. If four out of five frames were not identified as “occluded”, the actuator continued to extend until this criterion was met. Once “true occlusion” was reached from this process and identified by the connected flow sensor, the actuator held this occluded state for one minute to check for slippage or poor placement of the tourniquet, after which the motor was retracted and the user removed and disassembled the tourniquet, wherein a final 30s baseline was recorded.

During real-time testing, three different guidance protocols were evaluated and compared to test efficacy in finding a suitable point to begin occlusion. The first standard method deemed a site was suitable if the object detection AI model was able to find all three notable classes – the vein, artery and bone – and were relatively centered in the ultrasound image. The second method pooled predictions made on a set of consecutive ultrasound frames grabbed and input into the model. With this method the intention was to account for objects in view that caused inconsistent detections in between predictions ran through model inference. The last guidance method only tried to find one major vessel in frame in addition to the underlying bone under the premise that both vessels are close enough to one another that if one is in view the other is close enough for successful junctional occlusion. The three guidance protocols were tested to determine the time it took to trigger motor actuation for 10 iterations (*n* = 10). The best performing guidance protocol was integrated into both prototypes and tested in the JUGO phantom, as described above, in duplicative real-time test runs for FRejT and BaTS (*n* = 2 each).

### Statistical analysis

Different statistical analyses were performed depending on the comparison being made across the different experiments performed. However, it is worth noting that this study was proof-of-concept in nature and was not statistically-powered for assessing significant differences. To compare AI model performance between each Occlusion AI model, McNemar tests (NCSS 2025, Kaysville, UT, USA) were used to quantify differences between each occlusion AI model pair, where overall accuracy and inaccuracy vs. ground truth were assessed and when models agreed and disagreed. A p-value less than 0.05 indicated a statistically significant difference between compared models and results are indicated in the result where appropriate.

For all other comparisons (GraphPad Prism 10.6.1, La Jolla, CA, USA), we first assessed for normality by Shapiro-Wilk test, where p-value less than 0.05 indicated non-normality for the statistical analysis. When comparing RT testing performance between FRejT and BaTS, unpaired two-tailed Student’s T-Tests were used. ordinary one-way analysis of variance (ANOVA), post-hoc Holm-Šídák test was used when comparing overall times to occlusion for the prototypes and two commercial tourniquet products. A non-parametric Kruskal-Wallis test, post-hoc Dunn’s test was used to compare guidance timings and IOUs across prediction classes captured in real-time. For all analyses, p-values less than 0.05 denoted statistical significance and are indicated in the text and figures, along with the specific statistical test used.

## Data Availability

The datasets presented in this article are not readily available because they have been collected and maintained in a government-controlled database that is located at the U.S. Army Institute of Surgical Research. As such, data can be made available through the development of a business agreement with the corresponding author. Requests for the datasets should be directed to Dr. Eric J. Snider, eric.j.snider3.civ@health.mil.

## Declarations

### Disclaimer

The views expressed in this Article are those of the authors and do not reflect the official policy or position of the U.S. Army Medical Department, Department of the Army, DoD, or the U.S. Government.
